# Fibrosarcoma of the childhood mandible

**DOI:** 10.1186/1746-160X-4-21

**Published:** 2008-09-16

**Authors:** Martin Gosau, Florian G Draenert, Wolfgang A Winter, Jörg Mueller-Hoecker, Oliver Driemel

**Affiliations:** 1 Department of Cranio-Maxillo-Facial Surgery, University of Regensburg, Germany; 2 Department of Cranio-Maxillo-Facial Surgery, Ludwig Maximilians University of Munich, Germany; 3 Institute of Pathology, Ludwig Maximilians University of Munich, Germany

## Abstract

A case of low-grade intraosseous fibrosarcoma of the mandible in a 9-year-old girl is described. The patient underwent pre-surgical chemotherapy which was abandoned as unsuccessful after two cycles. Radical tumour resection and mandibular reconstruction with a titanium bar were performed 3 months after diagnosis. No adjuvant therapy was given and lymph node dissection was not performed. No signs of recurrences or metastasis have been observed after a follow up time of 3 years so far. This article is presented to document the rarity of fibrosarcomas in the jaws of children and emphasizes the possible changes in the appearance of radiological imaging under tumour progression.

## Introduction

Fibrosarcoma is a malignant neoplasm of mesenchymal origin. Its occurrence in the jaw especially in childhood is very rare. An infantile and an adult form are described showing identical histopathological features but differing in survival prognosis. The presented case describes an fibrosarcoma originating in the mandible of a young girl. Of special interest is the change in radiological appearance of the tumour during treatment.

### Clinical history

**Figure 1:**
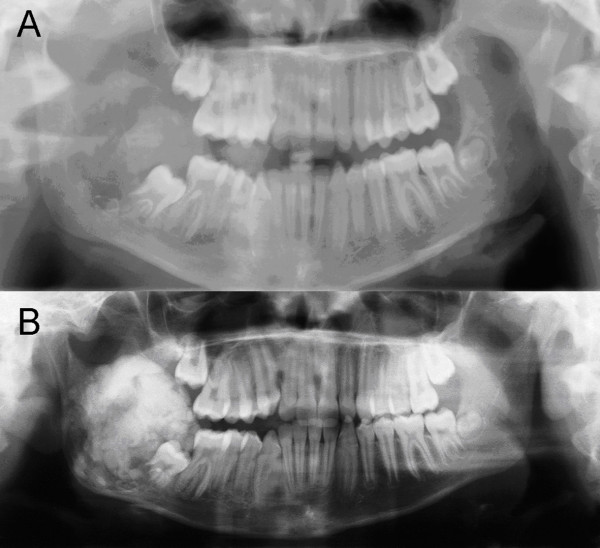
**A: Panoramic radiography at first presentation: A large radiolucent lesion with irregular borders infiltrating the right ramus of the mandible.** Displacement of tooth 47. B: Panoramic radiography after 3 months: The growth of the tumour mass is visible with a striking increase of radio-opaque structures.

**Figure 2:**
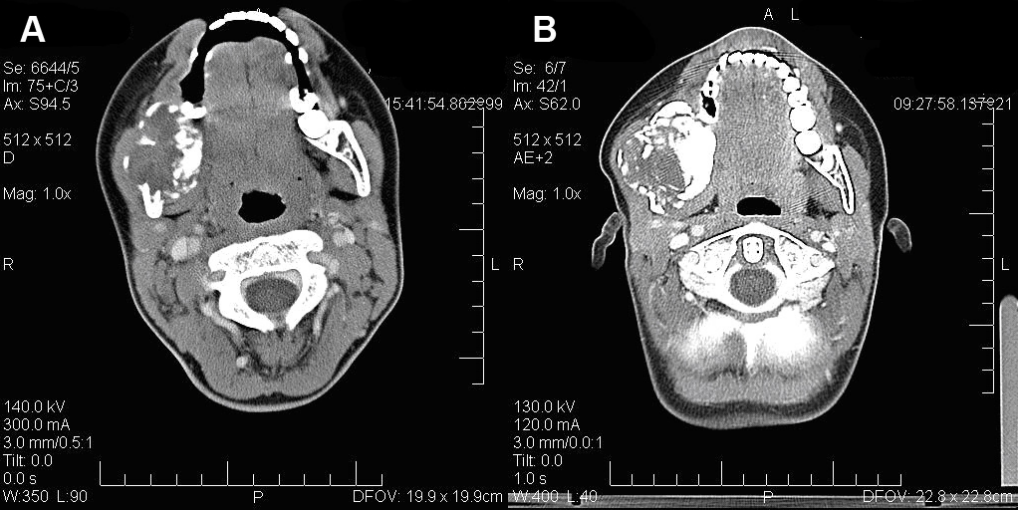
**Preoperative computed tomography scan in comparison to the scan at first admission, revealing the tumour progression.** A: CT before treatment: In the right mandibular angle a heterogenous mass of 3.0 × 3.6 cm is demonstrated, showing a rather sharp margin with cortical destruction. B: CT three months later: The computed tomography scan three months later reveals progression of the tumour mass (4.5 cm), with a further ballooning of the cortical structure and an increase of hard tissue, some osteolytic lesions and a central soft tissue component.

A 9-year-old girl was referred to the own department with a hard, slightly painful, well-circumscribed swelling (4 × 4 × 5 cm^3^) at the right mandibular angle, which was noticed by the parents 2 months before first presentation. There was no ulceration of the overlying mucosa. Mouth-opening was reduced to 32 mm due to pain. Paraesthesia was evident in the right lower lip. Radiological examinations with panoramic radiography (Figure. 1) and computed tomography scanning (Figure. 2) showed a large radiolucency with ill-defined borders located in the right mandibular angle and ramus. Histopathological findings revealed a low grade fibrosarcoma (FNCLCC Grading system: grade 1, pathology confirmed by Prof. Katenkamp, sarcoma reference centre, Jena and Prof. Jundt, bone tumour reference centre, Basel). Computed tomography of the chest showed a subpleural contrast-medium-enhancing structure (0.4 cm^3^). Pulmonary metastasis appeared to be a possible explanation for this, leading to initial treatment with neoadjuvant chemotherapy with cisplatin/doxorubicin in the first cycle and cisplatin/ifosphamide in the second cycle (according to the protocols Euramos-1/COSS and COSS 04). A computed tomography scan after the chemotherapy revealed no change in the initial pulmonary finding, whilst the primary tumour in the right mandibular angle showed progression and a striking gain of tumoural calcification (Figure. 1, 2). The structure in the lung was now considered to be post inflammatory rather than malignant and surgical resection of the mandibular tumour was planned.

**Figure 3:**
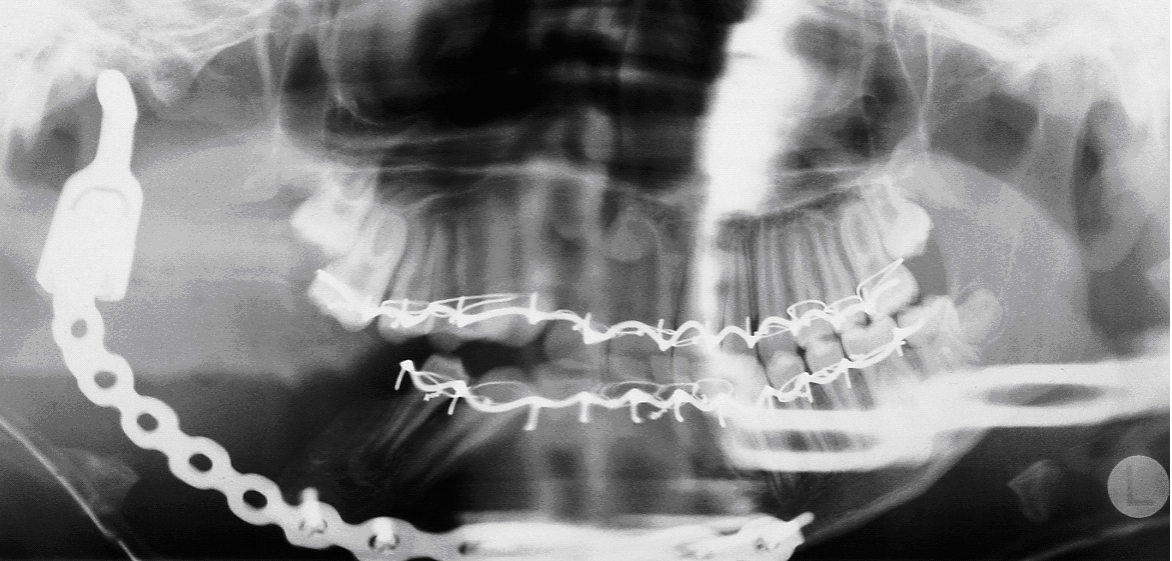
**Panoramic radiography post operation.** The combined Recontruction locking plate with artificial condylus can be seen.

**Figure 4:**
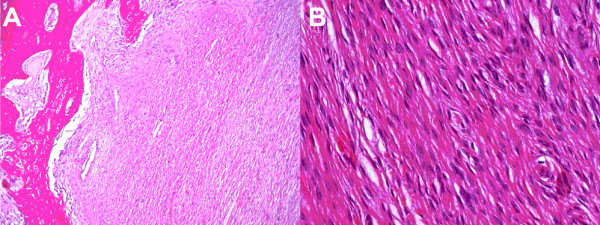
**Histopathological findings.** A: (HE staining, Magnification, obj. 10×) Histological sample from the margin of the tumour to show fibrous tumour tissue with newly built woven bone. B: (HE staining, Magnification, obj. 40×) Histological sample from the centre of the tumour to show the characteristic spindle-shaped cells. The associated fibres are arranged in interlacing bands or fascicles (Herring-bone pattern).

A hemimandibulectomy with exarticulation of the right condyle and temporary reconstruction with titanium osteosynthesis plate and condylar prosthesis was performed (Figure. 3). The histological examination of the resected tissues showed a tumour mass with a small number of spindle-shaped cells and proliferation of collagen fibres (Figure. 4). Destruction of cortical bone and new production of woven trabecular bone were visible (Figures. 4). Immunohistochemical examinations were consistently negative for actin, desmin, S 100-protein, Bcl 2 and cytokeratins 7 and 19. There was no evidence of an osteosarcoma since the formation of tumour osteoid was not a feature. A monophasic synovial sarcoma could also be ruled out by FISH-analysis, showing no X18 translocation.

3 Years after the primary operation the patient shows no sign of recurrence or metastasis. The pulmonary finding shows no change or growing.

## Discussion

Fibrosarcoma is a very rare malignancy with possible occurrence in the whole head and neck region [1]. It accounts only for one percent of all tumours in this region [2-4]. Due to location the more frequently seen periostal form is differentiated from the intraosseous fibrosarcoma. The periostal form shows a better overall 5-year survival rate of 75% [5]. The intraosseous form in the head and neck region mainly occurs in the mandible [3]. The WHO reports an overall 10-year survival of 83% in low grade and 34% in high grade sarcoma of the bone [6].

The mean age for the occurrence of fibrosarcoma is between the 2^th ^and 6^th ^decade with equal gender distribution [6]. Fibrosarcomas rarely occur before the 3^rd ^decade [7]. The adult fibrosarcoma is differentiated from the infantile type which occurs according to WHO-definition before the 3^rd ^year of age. Although infantile and adult fibrosarcoma are histologically identical, the infantile form carries a much more favourable prognosis. The infantile form meastasizes rarely and has a natural history similar to that of fibromatosis [4,6].

Typically, the tumour presents with swelling, associated with pain and paraesthesia [1,3,8]. Radiological imaging of fibrosarcomas reveals radiolucent lesions with a geographical, moth-eaten or permeative pattern of bone destruction [5,6,9]. The absence of tumoural calcification or ossification can be of importance in differentiating fibrosarcomas from other malignancies such as chondrosarcomas and osteosarcomas. In contrast, the change of appearance in radiographic imaging, in the presented case, is striking. Whilst the initial radiographic findings were a purely osteolytic process with ill-defined borders, the radiographic follow up after 3 months revealed a completely different pattern with a high degree of radio-opacity resulting from increased mineralisation of the tumour mass due to new bone formation under chemotherapy.

Fibrosarcomas are graded from low to high malignancy after the FNCLCC grading system, depending on the number of mitotic figures, tumour differentiation and the presence of tumour-necrosis [6,9,10].

The prognosis is highly dependent on the tumour-grading and the success of complete resection [1,2,4,6,11]. The need for adjuvant radiotherapy and/or chemotherapy is still unclear but there is normally an indication in high-grade tumours because these tumours may present with subclinical or microscopic metastases at the time of diagnosis. The need for prophylactic neck dissection is controversially discussed and it is not performed in all cases [3,12,13]. The presented case was treated with neoadjuvant chemotherapy, surgical resection and alloplastic mandibular reconstruction. Having regard to the age of the patient, the histological grade of the tumour and the absence of metastasis, it was decided that neither adjuvant therapy nor prophylactic neck dissection are indicated.

## Competing interests declaration

The authors declare that they have no competing interests.

## Authors' contributions

MG and FGD analysed the case, reviewed all patient data and drafted the manuscript. The operation planning and the operation were performed by WAW and MG. JM-H carried out the histological analysis and contributed to the writing of the final version. MG, FGD, WAW and OD contributed substantially to discussion and in writing of the paper. All authors reviewed the paper for content and contributed to the writing of the manuscript. All authors approved the final report.

## Consent section

Written informed consent was obtained from the patient for publication of this case report and accompanying images. A copy of the written consent is available for review by the Editor-in-Chief of this journal.

## References

[B1] Soares AB, Lins LHS, Mazedo AP, Neto JSP, Vargas PA (2006). Fibrosarcoma originating in the mandible. Med Oral Patol Oral Cir Bucal.

[B2] Leitner C, Hoffmann J, Kröber S, Reinert S (2007). Low-grade malignant fibrosarcoma of the dental follicle of an unerupted third molar without clinical evidence of any follicular lesion. J Craniomaxillofac Surg.

[B3] Pereira CM, Jorge J, Di Hipolito O, Kowalski LP, Lopes MA (2005). Primary intraosseous fibrosarcoma of the jaw. Int J Oral Maxillofac Surg.

[B4] Wanebo HJ, Koness RJ, MacFarlane JK, Eilber FR, Byers RM, Elias EG, Spiro RH (1992). Head and neck sarcoma: report of the head and neck sarcoma registry. Society of head and neck surgeons committee on research. Head Neck.

[B5] Theodorou DJ, Theodorou SJ, Sartoris DJ (2003). Primary non-odontogenic tumors of the jawbones. An overview of essential radiographic findings. J Clin Imag.

[B6] Kahn LB, Vigorita V, Fletcher CDM, Unni KK, Mertens F (2002). Fibrosarcoma of bone. World Health Organization Classification of Tumours Pathology and Genetics of Tumours of Soft Tissue and Bone.

[B7] Gorsky M, Epstein JB (1998). Head and neck and intra-oral soft tissue sarcomas. Oral Oncol.

[B8] Orhan K, Orhan AI, Oz U, Pekiner FN, Delibasi C (2007). Misdiagnosed fibrosarcoma of the mandible mimicking temporomandibular disorder: a rare condition. Oral Surg Oral Med Oral Pathol Oral Radiol Endod.

[B9] Rao BN, Santana VM, Fleming ID, Pratt CB, Shapiro D, Fontanesi J, Kumar APM, Austin BA (1989). Management and prognosis of head and neck sarcomas. Am J Surg.

[B10] Fletcher CDM, Sudaram M, Rydholm A, Coindre JM, Singer S, Fletcher CDM, Unni KK, Mertens F (2002). Soft tissue tumours: Epidemiology, clinical features, histopathological typing and grading. In World Health Organization Classification of Tumours. Pathology and Genetics of Tumours of Soft Tissue and Bone.

[B11] Daw NC, Mahmoud HH, Meyer WH, Jenkins JJ, Kaste SC, Poquette CA, Kun LE, Pratt CB, Rao BN (2000). Bone Sarcomas of the head and neck in children. Cancer.

[B12] Nagler RM, Malkin L, Ben-Arieh Y, Laufer D (2000). Sarcoma of the maxillofacial region, follow-up of 25 cases. Anticancer Res.

[B13] Yamaguchi S, Nagasawa H, Suzuki T, Fuji E, Iwaki H, Takagi M, Amagasa T (2004). Sarcomas of the oral and maxillofacial region: A review of 32 cases in 25 years. Clin Oral Investig.

